# The Book World of Medicine and Science

**Published:** 1907-11-23

**Authors:** 


### THE BOOK WORLD OF MEDICINE AND SCIENCE.

A TEXT-BOOK OF THE SCIENCE AND ART OF OBSTETRICS. By HENRY J. GARRIGUES, A.M., M.D. Late Professor of Obstetrics in the Post-Graduate School and Hospital; in the School for Clinical Medicine, New York. (Lippincott Co., Philadelphia and London. 1907. Pp. 870 and 525 illustrations. Price 25s.)

THE fact that this work has now reached its second edition speaks well for its popularity. It is an eminently practical work, and is written largely as a result of the author's experience, although due regard is paid to the opinions of others. Much new material has been added, notably on the subject of vaginal Cæsarean section, the toxæmia of pregnancy, Bossi's dilator, etc. We have always considered that this book is more suitable for the man in practice than the student, and we see no reason to alter our view. It is an eminently readable book, written in the somewhat colloquial style of our transatlantic brethren, and on this account will no doubt appeal to many.

THE NUTRITION OF MAN. By RUSSELL CHITTENDEN, Ph.D., LL.D., Sc.D. London. William Heinemann. 1907. 14s. net.

DR. CHITTENDEN has been well advised in publishing, in a cheap and popular form, the digest of his Lowell lectures on Nutrition delivered in the early part of the present year. His book is a valuable contribution to the complex subject of metabolism and dietetics which no physiologist, and, we may add, no medical man, should pass by without close attention to the practical conclusions drawn from a vast number of valuable experiments. Possibly, on first reading these lectures, the student of dietetics will say that they furnish nothing which is materially new. Habitual excess of food, over and above what is needed to meet the actual wants of the body, is not only uneconomical but distinctly disadvantageous. Cornaro, the pioneer of food reform in Europe, knew this truth as well as modern physiologists know it. But few experimenters have gone so fully into the question of low feeding as Professor Chittenden appears to have done. He has fed a number of dogs on the lowest possible proteid diet, and has proved conclusively that, under normal conditions, the animals do not lose weight, but gain flesh, on the insignificant diet scales which he provided for their consumption. Not content with experiments on animals, he has had the courage, so often lacking in physiological inquirers, to endeavour to obtain confirmatory data from human beings. The most interesting part of his conclusions, therefore, are those which deal with the effects of low diet upon men in active work. A group of eight University athletes, all large consumers of proteid food previous to the time when they consented to become subjects of experiment, had their daily consumption of proteid food gradually diminished, their total consumption of food being at the same time decreased. The experiment lasted five months, and during the last two months the average daily excretion of metabolised nitrogen of these eight subjects amounted to 8.81 grams per man. The general result seems to have been that the subjects unconsciously "drifted toward a simple vegetable diet," maintaining body-weight, health, physical strength, and perfect muscular tone. The excellent photographs with which the book is illustrated show the physical condition of these subjects of a low proteid diet very well, and they help considerably to an appreciation of the results of the experiments. The last chapter, dealing with the practical applications of the data furnished by these investigations, is worthy of careful study. One of these is the necessity of using salt when a purely vegetable diet is consumed, a matter which has previously been pointed out by Bunge. Chittenden argues that whenever there is a weakened condition of the kidneys there is reason for reducing the rate of proteid exchange to the lowest level consistent with the maintenance of equilibrium, ". . . thereby diminishing the amount of nitrogenous waste to be eliminated." He further suggests care and discrimination in the selection of a vegetable diet in these cases, so as to regulate the quantity of saline waste to be excreted by the kidneys, and contends that such care is as important in renal cases as the exclusion of carbohydrates in diabetes or glycosuria. "The master words"—to quote his own summary—"which promise to help in the carrying out of an intelligent plan of living are moderation and simplicity: moderation in the amount of food consumed daily, simplicity in the character of the dietary." These, in fact, are the conclusions which Cornaro supported, and they are those which experience has proved to be the best. For those who wish to know the scientific facts on which they are based, Professor Chittenden's interesting volume will serve as a clear and concise guide.

MEDICAL DIAGNOSIS. By C. L. GREENE, M.D., Professor of the Theory and Practice of Medicine in the University of Minnesota. Pp. 683, with seven coloured plates and 230 illustrations. Demy 8vo. (Published by Messrs. Rebman, Ltd., London. Price 13s. 6d. net.)

THE number of manuals and text-books of medicine already available for medical students and practitioners is so large that one would scarcely think there could be room for more. The volume before us has, however, several points about it which recommend it as a book likely to be of value to practitioners. It professes to have been written for students also; but if by students one understands young men who have yet to learn their medicine, we think they would do wrong to read this in place of such well-known text-books as those of Professor Osler or Dr. Frederick Taylor. The present volume is written with such terseness and with so little discussion that it is essentially suited for those who already know a good deal rather than for those who have still to learn almost all. It is too lengthy to be classed with cram-books; nor is it a mere compendium, for it bears the stamp of the author's personal experience and opinion; yet its style is distinctly summary and dogmatic, and the arrangement is very business-like and clear. It might be called a revision-book rather than a manual—a revision-book not only of symptoms and differential diagnosis, but also of physical signs and clinical methods of investigating cases generally. The text restricts itself to diagnosis, as the title implies; treatment is not discussed at all. The illustrations are small but good, and the coloured plates are excellent. Unproven or unessential theories and obsolete or superfluous methods are omitted. Wherever possible space has been economised, so that the maximum amount of useful information is given in the fewest possible words. The busy practitioner who cannot afford a large work of many volumes, but who wishes to have something by him to which he can quickly refer for some point he has forgotten, or to some method he is not quite sure about, will find this volume very useful. The volume is very heavy for its size, a heavily glazed paper having been found necessary on account of the illustrations and plates.

